# Improving Compliance With the Surgical Safety Checklist: A Quality Improvement Project at Almanagil Teaching Hospital, Sudan

**DOI:** 10.7759/cureus.95060

**Published:** 2025-10-21

**Authors:** Marwa Yousif, Ibrahim Adil Hamadelniel Alhadi, Elwathig Abdalla, Husham Siddig Ahmad Altayeb, Israa Awad Ahmed Mohamed, Mohanad Elsafi Mossaad Elbashier, Hashim Bashir Elmadani Ahmed, Mohamed Ziada, Ibrahim Hamad Ibrahim Hamad, Mohey Aldien Ahmed Elamin Elnour, Hala Fathi EmamElkhir Omer, Mohammad Alrawi, Ahmed Modawi Bakheet Jabraldar, Eman Abubakeralsideeg Diaaldeen Mohamed, Mohammed Ahmed Nasir Ahmed, Manasik M. Elmurtada Mubarak Ismail, Husam Eldin Abuelgassim Hassan Balila, Wafa Abdalla, Israa Isam Mohamed, Abubakr Muhammed

**Affiliations:** 1 General Surgery, Almanagil Teaching Hospital, Almanagil, SDN; 2 Orthopaedics, Almanagil Teaching Hospital, Almanagil, SDN; 3 Surgery, Almanagil Teaching Hospital, Almanagil, SDN; 4 Trauma and Orthopaedics, Almanagil Teaching Hospital, Almanagil, SDN; 5 General Surgery/Vascular Surgery, Almanagil Teaching Hospital, Almanagil, SDN; 6 Department of Biomedical Sciences, Qatar University, Doha, QAT

**Keywords:** al-managil teaching hospital, checklist, operative documentation, patient safety, quality improvement project, surgical safety

## Abstract

Background: The WHO Surgical Safety Checklist (SSC) is a globally recognized tool that reduces perioperative morbidity and mortality. However, adherence to SSC practices remains suboptimal in many low-resource settings.

Purpose: This project aimed to assess and improve compliance with the WHO SSC and operative documentation standards at Al Managil Teaching Hospital, Sudan.

Methods: A two-cycle quality improvement audit was conducted in July 2025. In the first cycle, 50 randomly selected operative notes were reviewed for SSC compliance. Interventions included staff training, standard documentation templates, visual reminders, and regular feedback. Another 50 randomly selected postoperative notes were reassessed after implementation. Data were analyzed using chi-square and Fisher’s exact tests, with p < 0.05 considered significant.

Results: Compliance improved across nearly all SSC domains. Pulse oximeter checks increased from 9 (18%) to 50 (100%), anesthesia machine and medication checks from 10 (20%) to 50 (100%), and allergy checks from 9 (18%) to 49 (98%) (all p < 0.001). Before incision, equipment issue documentation rose from four (8%) to 50 (100%), team introductions from 11 (22%) to 50 (100%), and timely antibiotic prophylaxis from 42 (84%) to 50 (100%) (p = 0.01). Demographic documentation (gender, unit, address) improved from 0 (0%) to 50 (100%). Only surgical site marking (from 54% to 62%) and risk checks (from 90% to 98%) showed a non-significant change.

Conclusion: Educational and structural interventions, including standardized templates and feedback, significantly enhanced SSC adherence and documentation quality. The project demonstrates that low-cost, sustainable quality improvement measures can foster a culture of surgical safety in resource-limited hospitals.

## Introduction

Safety and quality in surgery are essential values in contemporary perioperative care, with internationally recognized standards established to reduce preventable harm and improve patient outcomes. The Royal College of Surgeons of England (RCS) outlines such standards in Good Surgical Practice (2014) and its updated 2025 edition, emphasizing documentation, communication, teamwork, and adherence to safety protocols [1,2]. These recommendations complement the principles of Good Medical Practice by the General Medical Council, reflecting the ethical, legal, and professional duties required in surgical care.

The World Health Organization (WHO) introduced the Surgical Safety Checklist (SSC) in 2008 as part of its Safe Surgery Saves Lives program, marking a pivotal milestone in the history of perioperative safety. The checklist comprises three key phases - Sign In (before anesthesia induction), Time Out (before skin incision), and Sign Out (before the patient leaves the operating room) - each designed to verify patient identity, surgical site, allergy status, and critical team communication. Its implementation has been shown to significantly reduce avoidable errors and perioperative mortality worldwide [3,4].

Among the most important instruments for enhancing surgical safety is the WHO SSC. This globally endorsed tool has been shown to significantly reduce perioperative morbidity and mortality by introducing structured safety checks at critical points of the surgical process [3,4]. However, effective implementation in low-resource settings such as Sudan is challenged by factors such as high staff turnover, limited availability of standardized forms, and inconsistent training, necessitating localized quality improvement approaches. Despite its proven effectiveness, compliance with checklist protocols and operative documentation remains suboptimal in many low-resource settings, exposing patients to preventable risks and weakening medico-legal accountability [5]. A growing body of evidence highlights the role of audit and feedback in strengthening adherence to surgical safety practices. Studies from tertiary hospitals in Lahore, Pakistan, and Bangalore, India, demonstrated that targeted staff education and structured intervention programs improved both operative note documentation and compliance with RCS guidelines [6,7]. Similar findings have been reported in Sudan, where audits and educational initiatives at Dongola, Port Sudan, and Elobeid Teaching Hospitals led to marked improvements in documentation standards and checklist adherence [8-11].

Drawing on this evidence, the current quality improvement project (QIP) was conducted at Almanagil Teaching Hospital, Almanagil, Sudan, in July 2025. The hospital was selected because baseline observations revealed inconsistent completion of the WHO SSC, absence of standardized operative note templates, and variable documentation practices across surgical teams. As a regional referral center serving a large rural population with limited resources, Almanagil Teaching Hospital provided a representative setting to assess and address these gaps. The project aimed to assess and enhance adherence to the WHO SSC and operative documentation standards through audit-based evaluation and targeted staff interventions. By fostering a stronger culture of safety, this initiative seeks to improve perioperative care and strengthen surgical practice within the Sudanese healthcare system.

## Materials and methods

The quality improvement project (QIP) was conducted at Al-Managil Teaching Hospital in July 2025 to enhance compliance and consistency in the application of the World Health Organization (WHO) Surgical Safety Checklist (SSC). The project followed a prospective audit design comprising three phases: pre-intervention evaluation, intervention, and post-intervention evaluation.

Pre-intervention phase

A retrospective review of 50 randomly selected operative notes was conducted to establish baseline adherence to SSC documentation. These notes were drawn from general surgery operations performed between June and July 2025, including both elective and emergency procedures. Selection was based on the availability of complete operative records within the study period, ensuring representative coverage of typical surgical workloads. All notes originated from the general surgery department, which was chosen as it performs the majority of procedures in the hospital and serves as the primary training and service unit.

The notes were assessed for completion of essential SSC parameters, such as patient identifiers, operative details, team members’ signatures, intraoperative checks, and postoperative instructions. The review revealed several deficiencies, particularly in documenting patient details, team sign-offs, and pre-anesthetic verifications. The audit was designed to proceed regardless of the baseline level of compliance to allow measurement of improvement following intervention.

Common issues included incomplete checklist steps during emergencies, illegible documentation, and inconsistent team participation, reflecting weak collective ownership of the safety process.

Root cause analysis

A root cause analysis discovered a number of factors that led to incomplete or unreliable documentation. These were ignorance of the entire scope of RCS and WHO checklist mandates, inadequate documentation templates, time constraints due to workload, and inadequate feedback systems. In addition, the new employees were not informed and trained in a consistent manner, which restricted their compliance with the standards of documentation.

Intervention phase

According to the preliminary results, specific interventions were introduced to overcome the detected gaps. In line with existing hospital policy, the intervention focused on enforcing and supporting the consistent application of the WHO Surgical Safety Checklist (SSC) in all surgical procedures through structured supervision, feedback, and team accountability.

**Figure 1:**
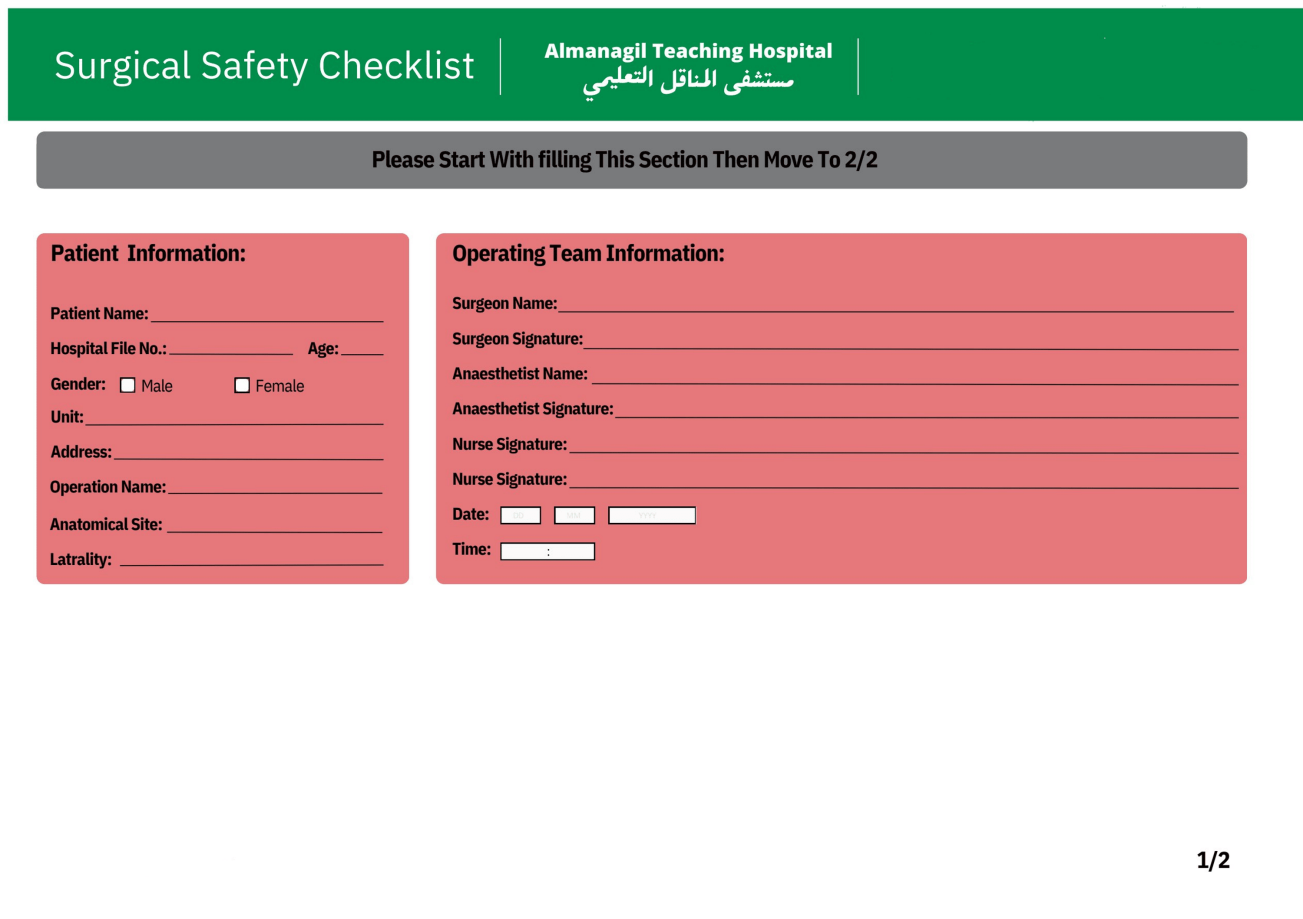
Page One of the New Safety Surgical Checklist

**Figure 2:**
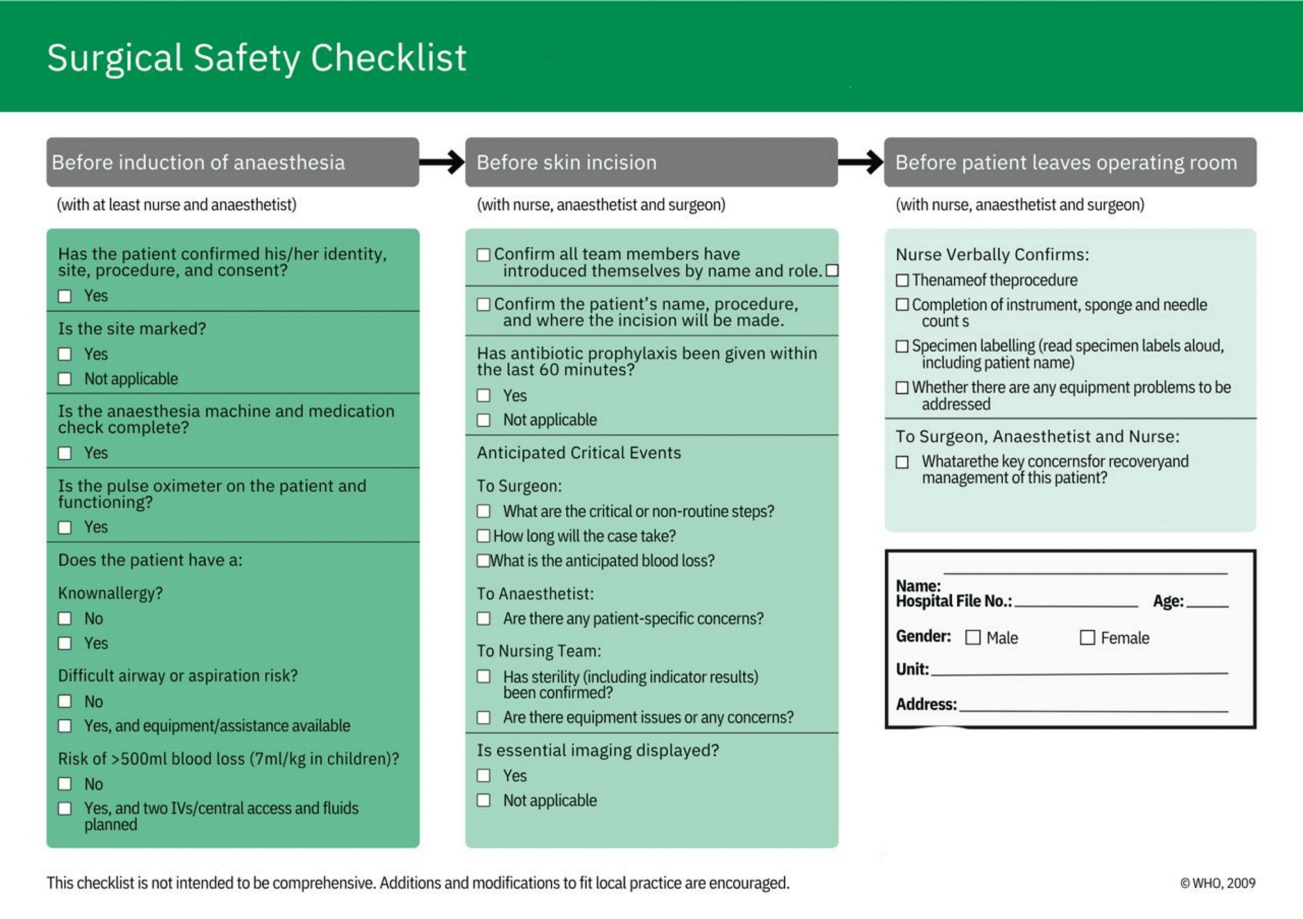
Second Page of the New Safety Surgical Checklist

The interventions were applied across both elective and emergency operations to ensure uniform adherence to checklist procedures regardless of case urgency. Templates of standardized documentation were also implemented to ensure uniformity in operative notes (Figures 1, 2). The design of these templates was guided by the deficiencies identified in the pre-intervention audit, ensuring that all under-documented parameters were explicitly incorporated.

The full WHO Surgical Safety Checklist was maintained without omitting any items; however, additional context-specific fields - such as patient address, consultant name, and postoperative plan - were added to address documentation gaps revealed in Cycle 1.

A structured educational program was delivered to all members of the surgical team, including surgeons, anesthetists, and theater nurses. It consisted of two interactive workshops and one focused bedside teaching session aimed at reinforcing checklist use, improving communication, and ensuring complete documentation.

Comprehension and readiness were verified through immediate post-training quizzes and supervised checklist simulations, ensuring that participants demonstrated correct understanding before resuming operative duties. Posters and checklists as visual reminders were placed in the operating theaters, and continuous auditing feedback was communicated with the surgical teams. Senior management enhanced oversight and guidance for junior physicians to strengthen compliance with best practices in documentation and surgical safety.

Post-intervention phase

One month after implementing the interventions, a second audit cycle was conducted to assess the impact of the quality-improvement measures. Operative notes were re-examined using the same data-collection checklist and evaluation criteria as in the pre-intervention phase to ensure methodological consistency. Fifty randomly selected operative notes from surgeries performed during the follow-up month were reviewed for adherence to all WHO SSC parameters and operative-note components.

This phase focused solely on the procedural reassessment and documentation of compliance rather than on statistical outcomes, which are presented separately in the Results section.

Each parameter was evaluated for completeness, legibility, and accuracy according to the standardized template introduced during the intervention. Findings from this phase provided the basis for comparing pre- and post-intervention performance and planning subsequent reinforcement and re-audit cycles.

Data analysis

Data were analyzed using descriptive and inferential statistical methods. Compliance with each parameter of the SSC was expressed in terms of frequencies and percentages to provide a clear picture of documentation patterns in both the pre- and post-intervention phases. Comparative analysis was conducted to evaluate the effect of the interventions by measuring improvements in compliance rates across the two cycles. The chi-square test was applied, as appropriate, to assess the statistical significance of differences between the baseline and re-audit findings, with a p-value of <0.05 considered statistically significant. This approach allowed not only the identification of overall improvements but also highlighted specific domains where compliance remained suboptimal and warranted further reinforcement.

Ethical considerations

Ethical approval for the project was obtained from the hospital administration and the Almanagil Teaching Hospital Research and Ethics Committee. As this project was categorized as a local quality improvement audit, a formal IRB number was not issued. Since the study involved a retrospective review and audit of operative notes without patient identifiers, issues of confidentiality and privacy were strictly maintained. All data were anonymized before analysis, and no personal identifiers were included in reports or publications. The project adhered to the principles of the Declaration of Helsinki and was conducted with the primary aim of improving patient safety and quality of care, without exposing patients to additional risks.

## Results

The SSC items at all steps of the surgical process improved significantly between the first and second audit cycles.

Before induction of anesthesia

Compliance with key safety checks increased markedly. Pulse oximeter functioning improved from 9 (18%) to 50 (100%); anesthesia machine and medication checks from 10 (20%) to 50 (100%); allergies checked from 9 (18%) to 49 (98%); difficult airway/aspiration risk checked from 9 (18%) to 49 (98%); and IV/fluids planning from 10 (20%) to 40 (80%). Patient confirmation of ID, surgical site, procedure, and consent rose from 22 (44%) to 50 (100%) (χ² = 36.16; p < 0.001). Surgical site marking (27 (54%) to 31 (62%)) and risk checking (45 (90%) to 49 (98%)) showed smaller, non-significant changes as both parameters had high baseline compliance prior to the intervention.

Before skin incision

Improvements were observed across nearly all domains. Nursing equipment issues increased from 4 (8%) to 50 (100%) (χ² = 81.52; p < 0.001). Discussion of critical roles and non-routine steps rose from 6 (12%) to 50 (100%) (χ² = 75.04; p < 0.001); case duration discussion from 9 (18%) to 50 (100%) (χ² = 66.14; p < 0.001); and team introductions by name and role from 11 (22%) to 50 (100%) (χ² = 60.70; p < 0.001). Sterility confirmation by the nursing team increased from 11 (22%) to 50 (100%), and anaesthesia team concerns discussed rose from 12 (24%) to 50 (100%) (both p < 0.001). Patient identity, procedure, and site confirmation improved from 15 (30%) to 50 (100%), while essential imaging display rose from 18 (36%) to 50 (100%) (both p < 0.001). Anticipated blood loss discussion increased from 28 (56%) to 50 (100%) (p < 0.001). Antibiotic prophylaxis within 60 minutes improved from 42 (84%) to 50 (100%) (χ² = 6.66; p = 0.010).

Operating team information

There were substantial gains in documentation accuracy and completeness. Second nurse signature increased from 0 (0%) to 50 (100%); anaesthetist signature from 1 (2%) to 50 (100%); nurse signature from 10 (20%) to 50 (100%); and surgeon signature from 30 (60%) to 50 (100%) (all p < 0.001). Minimal change was observed for parameters with already high baseline compliance, such as date, time, and team names.

Patient information

Documentation of patient identifiers improved dramatically. Gender, unit, and address each rose from 0 (0%) to 50 (100%) (all p < 0.001). Hospital file number improved from 7 (14%) to 50 (100%), laterality from 10 (20%) to 47 (94%), and anatomical site documentation from 22 (44%) to 48 (96%) (all p < 0.001). Patient age (47 (94%) to 50 (100%)) and operation name (47 (94%) to 50 (100%)) showed smaller, non-significant improvements.

Sign out

End-of-surgery processes also demonstrated significant improvement. Equipment issues addressed increased from four (8%) to 50 (100%); verbal confirmation of procedure name from nine (18%) to 50 (100%); instrument, sponge, and needle counts from 12 (24%) to 50 (100%); and specimen labeling/confirmation from 12 (24%) to 50 (100%) (all p < 0.001). Recovery and management concerns were discussed more consistently, improving from 33 (66%) to 50 (100%) (p < 0.001) (Table 1).

**Table 1:** Compliance with the Surgical Safety checklist parameters before and after intervention at Almanagil Teaching Hospital (August 2025) This table presents the comparison of compliance rates with World Health Organization Surgical Safety checklist parameters across two audit cycles (First Cycle = pre-intervention, n = 50; Second Cycle = post-intervention, n = 50). Compliance is reported as the frequency (percentage) of operative notes meeting each parameter. Statistical significance of improvements was assessed using the chi-square test (or Fisher’s exact test where appropriate), with a p-value of <0.05 considered significant. NA indicates that the statistical test could not be applied due to complete compliance in both cycles.

Section	Parameter	First Cycle (n=50)	Second Cycle (n=50)	Chi-Square	p-value
Before Induction of Anaesthesia	Pulse oximeter functioning	9 (18%)	50 (100%)	66.14	<0.001
	Anaesthesia machine & meds check	10 (20%)	50 (100%)	63.38	<0.001
	Allergy checked	9 (18%)	49 (98%)	62.44	<0.001
	Difficult airway/aspiration risk checked	9 (18%)	49 (98%)	62.44	<0.001
	IV/fluids planned	10 (20%)	40 (80%)	33.64	<0.001
	Patient confirmed ID/site/procedure/consent	22 (44%)	50 (100%)	36.16	<0.001
	Surgical site marked	27 (54%)	31 (62%)	0.37	0.5433
	Risk checked	45 (90%)	49 (98%)	1.6	0.206507
Before Skin Incision	Nursing equipment issues	4 (8%)	50 (100%)	81.52	<0.001
	Critical roles/non-routine steps	6 (12%)	50 (100%)	75.04	<0.001
	Case duration discussed	9 (18%)	50 (100%)	66.14	<0.001
	Team introductions by name/role	11 (22%)	50 (100%)	60.7	<0.001
	Nursing team sterility confirmed	11 (22%)	50 (100%)	60.7	<0.001
	Anaesthesia team concerns	12 (24%)	50 (100%)	58.11	<0.001
	Patient name/procedure/site confirmed	15 (30%)	50 (100%)	50.81	<0.001
	Essential imaging displayed	18 (36%)	50 (100%)	44.16	<0.001
	Anticipated blood loss discussed	28 (56%)	50 (100%)	25.7	<0.001
	Antibiotics prophylaxis within 60 min	42 (84%)	50 (100%)	6.66	0.009873
Operating Team Information	Second nurse signature	0 (0%)	50 (100%)	96.04	<0.001
	Anaesthetist signature	1 (2%)	50 (100%)	92.2	<0.001
	Nurse signature	10 (20%)	50 (100%)	63.38	<0.001
	Surgeon signature	30 (60%)	50 (100%)	22.56	<0.001
	Date provided	48 (96%)	50 (100%)	0.51	0.475051
	Anaesthetist name	49 (98%)	50 (100%)	0	1
	Surgeon name	50 (100%)	50 (100%)	NA	1
	Time provided	50 (100%)	50 (100%)	NA	1
Patient Information	Patient gender	0 (0%)	50 (100%)	96.04	<0.001
	Patient unit	0 (0%)	50 (100%)	96.04	<0.001
	Patient address	0 (0%)	50 (100%)	96.04	<0.001
	Hospital file number	7 (14%)	50 (100%)	71.97	<0.001
	Laterality stated	10 (20%)	47 (94%)	52.88	<0.001
	Anatomical site documented	22 (44%)	48 (96%)	29.76	<0.001
	Patient age filled	47 (94%)	50 (100%)	1.37	0.241028
	Operation name entered	47 (94%)	50 (100%)	1.37	0.241028
	Patient name recorded	50 (100%)	49 (98%)	0	1
Sign Out	Equipment issues addressed	4 (8%)	50 (100%)	81.52	<0.001
	Procedure name verbally confirmed	9 (18%)	50 (100%)	66.14	<0.001
	Instrument/sponge/needle count	12 (24%)	50 (100%)	58.11	<0.001
	Specimen labeled & confirmed	12 (24%)	50 (100%)	58.11	<0.001
	Recovery & management concerns discussed	33 (66%)	50 (100%)	18.14	<0.001

## Discussion

The observed improvements across checklist domains suggest that structured interventions - such as staff training, standardized documentation templates, and regular feedback - directly enhanced adherence to the WHO SSC and improved perioperative communication. These results indicate that even modest, low-cost measures can significantly influence behavior and teamwork within operating rooms. The marked increase in compliance is likely explained by three interrelated factors: (1) improved staff awareness through education, (2) workflow standardization that minimized ambiguity in documentation, and (3) a feedback mechanism that reinforced accountability among team members.

These findings reflect behavioral reinforcement rather than procedural change, showing that, when clinical teams understand both the rationale and expectations behind checklist use, compliance becomes more consistent and durable.

The findings align with international evidence on the success of checklist implementation. Krstulović et al. reported significant increases in SSC compliance following educational and organizational interventions in a tertiary hospital, where embedding structured safety protocols into daily practice proved essential [3]. Similarly, Alsadun et al. highlighted that routine checklist use not only reduces adverse surgical outcomes but also enhances team engagement and fosters greater trust in safety processes [4]. In our context, the intervention validated that structured education and visual reinforcement can produce similar effects even in resource-constrained hospitals.

Particular attention should be drawn to the improvements in patient-information recording and documentation of operating-team members. Accurate identification, operative details, and formal signatures improve accountability and strengthen medico-legal protection. These outcomes reflect the principles of Good Surgical Practice as outlined by the Royal College of Surgeons of England, which emphasizes documentation as a cornerstone of safe surgical care [1,2]. The positive documentation trends observed mirror the behavior-based improvements noted in previous audits from Bangalore, India, and Lahore, Pakistan [6,7].

Within Sudan, comparable initiatives in Dongola, Port Sudan, and Elobeid Teaching Hospitals have also demonstrated parallel gains in operative documentation and SSC compliance [8-11]. The present project extends this regional evidence, confirming that a culture of surgical safety can be strengthened through simple, context-appropriate strategies.

Beyond improving compliance, SSC interventions influence broader cultural change within surgical teams. Haynes et al. showed that checklist adoption across diverse hospitals worldwide not only reduced morbidity and mortality but also improved perceptions of teamwork [12]. Similarly, Brima et al. found that consistent checklist use fosters a sense of shared responsibility, empowering junior staff to communicate concerns and enhancing the overall safety climate [13]. These findings support the hypothesis that the SSC acts as both a clinical and cultural instrument for patient safety, particularly valuable in hierarchical, low-resource environments.

The sustainability of these gains depends on institutional commitment and continuous reinforcement. Studies from Sub-Saharan Africa emphasize that checklist interventions yield the greatest impact when combined with leadership support, regular monitoring, and integration into existing hospital policies [14]. Moreover, Molina et al. noted that surgical safety initiatives are more resilient when championed by local leaders who maintain ongoing training and mentorship [15]. In the context of Sudan, such localized ownership is crucial to ensure that improvements become routine practice rather than temporary outcomes.

Despite these encouraging outcomes, some parameters - most notably surgical-site marking - remained below optimal levels. This highlights the persistent challenges of translating checklist prompts into consistent clinical behavior, especially for items perceived as less critical when baseline compliance is already high. Targeted reinforcement, real-time observation, and repeated audit cycles will be necessary to sustain and advance these gains.

Limitations

While the results demonstrate strong improvement in checklist compliance, several limitations must be acknowledged. First, the study evaluated documentation rather than direct behavioral adherence; hence, it cannot confirm that all checklist steps were verbally performed during surgery. Second, the possibility of a Hawthorne effect exists, as staff awareness of ongoing auditing may have temporarily influenced behavior. Third, the short follow-up period limits conclusions about long-term sustainability, which will be re-evaluated in the planned third audit cycle. Fourth, as a single-center initiative, the generalizability of these findings to other institutions may be limited by contextual differences in resources and leadership engagement. Despite these constraints, the project successfully demonstrates a scalable, low-cost model for improving surgical documentation and safety compliance in similar low-resource environments.

## Conclusions

This quality improvement project confirmed that structured audit, education, and feedback effectively enhanced adherence to the WHO SSC at Almanagil Teaching Hospital. A third audit cycle is planned in six months to evaluate the sustainability of these gains and to reinforce parameters such as surgical-site marking. The combined model of education, standardized tools, and audit feedback represents a practical, low-cost approach to strengthening surgical safety in resource-limited hospitals.
