# Correction: Improving Compliance With the Surgical Safety Checklist: A Quality Improvement Project at Almanagil Teaching Hospital, Sudan

**DOI:** 10.7759/cureus.c392

**Published:** 2026-01-09

**Authors:** Marwa Yousif, Ibrahim Adil Hamadelniel Alhadi, Elwathig Abdalla, Husham Siddig Ahmad Altayeb, Israa Awad Ahmed Mohamed, Mohanad Elsafi Mossaad Elbashier, Hashim Bashir Elmadani Ahmed, Mohamed Ziada, Ibrahim Hamad Ibrahim Hamad, Mohey Aldien Ahmed Elamin Elnour, Hala Fathi EmamElkhir Omer, Mohammad Alrawi, Ahmed Modawi Bakheet Jabraldar, Eman Abubakeralsideeg Diaaldeen Mohamed, Mohammed Ahmed Nasir Ahmed, Manasik M. Elmurtada Mubarak Ismail, Husam Eldin Abuelgassim Hassan Balila, Wafa Abdalla, Israa Isam Mohamed, Abubakr Muhammed

**Affiliations:** 1 General Surgery, Almanagil Teaching Hospital, Almanagil, SDN; 2 Orthopaedics, Almanagil Teaching Hospital, Almanagil, SDN; 3 Surgery, Almanagil Teaching Hospital, Almanagil, SDN; 4 Trauma and Orthopaedics, Almanagil Teaching Hospital, Almanagil, SDN; 5 General Surgery/Vascular Surgery, Almanagil Teaching Hospital, Almanagil, SDN; 6 Department of Biomedical Sciences, Qatar University, Doha, QAT

The affiliation for Wafa Abdalla has been corrected to: Biomedical Sciences Department, Qatar University, Doha, Qatar.

